# Climate emergency and the food system: the impact of May floods on the community food environment of the Rio Grande do Sul State, Brazil

**DOI:** 10.1590/0102-311XEN130824

**Published:** 2025-05-26

**Authors:** Lauren Yurgel da Silva, Elma Izze da Silva Magalhães, Júlio Celso Borello Vargas, Larissa Loures Mendes, Raquel Canuto

**Affiliations:** 1 Programa de Pós-graduação em Alimentação, Nutrição e Saúde, Universidade Federal do Rio Grande do Sul, Porto Alegre, Brasil.; 2 Programa de Pós-graduação em Planejamento Urbano e Regional, Universidade Federal do Rio Grande do Sul, Porto Alegre, Brasil.; 3 Departamento de Nutrição, Universidade Federal de Minas Gerais, Belo Horizonte, Brasil.

**Keywords:** Food Social Space, Climate Change, Floods, Espacio Social y Comida, Cambio Climático, Inundaciones

## Abstract

The objective of this study was to describe the impact of the May 2024 floods on the community food environment of Rio Grande do Sul State, Brasil. This is an ecological study in the 92 municipalities of Rio Grande do Sul State that had at least one establishment that sells food affected by the floods. The geographic data on the flooding were extracted from the database of the Institute of Hydraulic Research of Federal University of Rio Grande do Sul and the information on food establishments was obtained from the database of the Special Secretariat of the Federal Revenue of Brazil (year 2022). Proportions of groups/categories of affected establishments were calculated and compared using Pearson’s chi-square test. Of the total number of establishments in the municipalities, 15.7% were located in the flooded area. In addition, 11 cities had more than 40% of their businesses flooded. Regarding the proportion of affected establishments in relation to the total number of establishments in each group/category of establishments, the group of establishments that sell in natura or minimally processed foods (17.1%) was the most affected (p < 0.0001); and fishmongers (28.5%) and restaurants (17.6%) were the most affected categories of in natura or minimally processed and mixed groups, respectively (p < 0.0001). In conclusion, a major impact of the floods on the food trade in Rio Grande do Sul State was observed, especially in establishments that sell in natura or minimally processed foods, denoting the importance of monitoring the reestablishment of the community food environment over time to ensure food security for the population.

## Resumen

El objetivo de este estudio fue describir el impacto de las inundaciones de mayo de 2024 en las tiendas de alimentos comunitarias de Rio Grande do Sul (Brasil). Se trata de un estudio ecológico en los 92 municipios de Rio Grande do Sul que tenían al menos una tienda donde se vende alimentos y que fue afectada por las inundaciones. Los datos geográficos de la inundación se obtuvieron de la base de datos del Instituto de Investigación Hidráulica de la Universidad Federal del Rio Grande do Sul; y la información de las tiendas donde se venden alimentos se obtuvo de la base de datos de la Secretaría Especial de Hacienda Federal de Brasil (año 2022). Las proporciones de grupos/categorías de tiendas afectadas se calcularon y compararon mediante la prueba de chi-cuadrado de Pearson. Del total de las tiendas de los municipios, el 15,7% estaban situadas en la zona inundada. Además, 11 ciudades tuvieron más del 40% de sus tiendas inundadas. En cuanto a la proporción de tiendas afectadas en relación al número total de tiendas en cada grupo/categoría de tiendas, el grupo de tiendas donde se venden alimentos *in natura* o mínimamente procesados (17,1%) fue el más afectado (p < 0,0001); y pescaderías (28,5%) y restaurantes (17,6%) fueron las categorías más afectadas de grupos *in natura* o mínimamente procesados y mixtos, respectivamente (p < 0,0001). Se concluye que hubo un gran impacto de las inundaciones en las tiendas de alimentos en Rio Grande do Sul, especialmente en los sitios donde se venden alimentos *in natura* o mínimamente procesados, lo que muestra la importancia de monitorear la restauración de las tiendas de alimentos lo largo del tiempo para garantizar la seguridad alimentaria de la población.

## Introduction

Brazil has experienced several environmental disasters in the last decade, including the ruptures of the Mariana, Minas Gerais State (2015) and Brumadinho (2019) ore tailings dams ^1^. But it was in May 2024 that Rio Grande do Sul, Brazil’s southernmost state with about 11 million inhabitants, faced one of the most significant climate disasters in the country, with 95% of its municipalities affected by the biggest floods in its history. Approximately 2 million people have been affected, 581,638 have been displaced, 800 have been injured, and 178 deaths have been confirmed ^2^. 

Floods are a direct consequence of the overheating of the oceans, which has altered rainfall patterns in the state, increasing the frequency of storms and the volume of precipitation ^3^. This type of extreme weather event is ever more frequent, and causes several damages, including the disruption of food systems ^4^ ^,^ ^5^ ^,^ ^6^ ^,^ ^7^. The food system refers to a set of elements and activities related to the production, processing, distribution, preparation, and consumption of food and the results of these activities ^8^. Food environment can be defined as the consumer interface with the food system, which encompasses the availability, affordability, convenience, promotion, quality and sustainability of food and beverages in wild, cultivated and built spaces, influenced by the sociocultural, political environment and the ecosystems in which they are inserted ^9^. The community food environment is one of the dimensions of the food system and concerns the availability (distribution, number and type) and accessibility of commercial food establishments ^10^. 

The community food environment can be particularly affected by floods due to the damage caused to the infrastructures and physical spaces of establishments in which food is produced, stored and sold ^4^, thus reducing the availability of food products and hindering the population’s physical and economic access to various types of food ^6^ ^,^ ^7^. This scenario contributes to the increase in food and nutrition insecurity, reducing the supply of in natura or minimally processed foods and favoring the consumption of ultra-processed foods, especially among vulnerable populations ^11^ ^,^ ^12^. Such factors are at the heart of the global syndemic, constituted by the pandemics of malnutrition, obesity, and climate change, which interact synergistically and share common social determinants, characterizing a challenging panorama on a global scale ^12^.

Given that extreme weather events can impact on ensuring food and nutritional security for affected populations, it is imperative to assess the impact of floods on the community food environment. Therefore, this study aimed to describe the impact of the floods that occurred in Rio Grande do Sul in May 2024 on the food retail in the affected municipalities.

## Methods

This is an ecological study, that had as its unit of analysis municipalities in Rio Grande do Sul State that had their food retail affected by the floods of May 2024. The State of Rio Grande do Sul, located in the southern region of the country, has a geographical area of 281,707.15km^2^, with 497 municipalities and a population of 10,882,965 inhabitants ^13^. The sample analyzed in the present study was composed of municipalities that had at least one establishment that sells food affected by the floods, that is, that was located in the flood range observed by geospatial analysis. From this sample, a subsample of the municipalities that had a proportion of affected food retail higher than the average percentage observed in the total sample was analyzed, to better understand the impact of the flood in the areas with a higher proportion of affected food retail. This subsample was selected by calculating the average percentage of food retail affected from the total sample and then identifying those municipalities whose percentage was higher than this value.

The geographic data of the flood were extracted from the database of the Institute of Hydraulic Research at Federal University of Rio Grande do Sul (UFRGS, acronym in Portuguese), with public access on the platform *Floods in Rio Grande do Sul − Database and geographic information in the Hydrographic Region of Lake Guaíba and Lagoa dos Patos in 2024*, by ArcGIS StoryMaps ^14^. The maps available show the spots in the flood area in the hydrographic region of lake Guaíba and Lagoa dos Patos, captured by satellite from the Sentinel-2 sensor on May 6 to 8, 2024. 

The information on the food retail was obtained from the database of the Special Department of Federal Revenue of Brazil (RFB, acronym in Portuguese), which condenses the data of active Brazilian Nationa Registry of Legal Entities (CNPJ, acronym in Portuguese), referring to the year 2022. The database contained information about the address, name, size of the company, main type of activity carried out and classification according to the National Classification of Economic Activities (CNAE, acronym in Portuguese). This classification derives from a standardization of commercial activities carried out by means of codes, which totaled nineteen different types of establishments. With the addresses, the geographic coordinates were extracted through the Google Maps georeferencing API (https://www.google.com/maps/), via the Awesome Table − Talarian extension (https://awesome-table.com/), a free extension that enables communication between the georeferencing API and a Google Sheets (https://workspace.google.com/products/sheets/) spreadsheet. The correction of georeferencing errors was performed using the software for geographic information systems QGIS 3.34.7 (https://qgis.org/en/site/), in which the points with errors were visualized and corrected by means of new georeferencing.

The establishments were grouped according to the methodology proposed by the Interministerial Chamber of Food and Nutrition Security ^15^, which classifies establishments according to the extent and purpose of processing the foods most frequently purchased by the Brazilian population in each category of establishment. This methodology derives from the Nova classification ^16^. The establishments were then classified into the following groups: (1) establishments that mainly sell in natura or minimally processed foods, such as fruit and vegetables, butchers and fishmongers; (2) establishments that predominantly sell ultra-processed foods, such as bars, snack bars, convenience stores, and candy stores; and (3) mixed establishments, which sell a mixture of other food categories, such as bakeries and restaurants. Supermarkets and hypermarkets were analyzed separately due to the wide variety of foods sold and the lack of consensus in the literature on their real influence on the population’s purchase profile ^17^. 

Through geospatial analysis in QGIS 3.34.7, the interpolation of the flood areas and the georeferencing of food retail was carried out, and those food establishments that were within the flood area were considered as affected. 

Statistical analyses were conducted using Stata software, version 14.0 (https://www.stata.com). The data analyzed were described by means of absolute and relative frequencies, considering the concepts of category of establishments (types of establishments, such as butchers, snack bars, restaurants, supermarkets etc.) and groups of establishments according to the Nova classification (in natura or minimally processed, mixed and ultra-processed). Three different measures were calculated regarding the proportion of categories of establishments affected: in relation to the total number of establishments; in relation to the total number of affected establishments in the group; and in relation to the total number of establishments in the same category. 

 In addition, the percentage of census tracts (unit of analysis) classified as food deserts in the cities whose food establishments were affected was identified as follows: the density of healthy establishments (in natura and minimally processed, mixed, and supermarkets and hypermarkets) per 10,000 inhabitants was calculated. Those sectors with a density of less than or equal to the 25th percentile were classified as food deserts ^15^. Pearson’s chi-square test was used to compare the proportions analyzed, considering a significance level of 5%. 

For the visual representation of the impact of the flood on the food retail, the percentages of affected establishments by category in each affected municipality were represented on maps with color grading, using five-tone Jenks Breaks, in which lighter tones indicate a lower impact of the flood, and darker tones represent a greater impact. 

## Results

Of the 497 municipalities in the state, 191 were affected by the flood and 92 had their food retail affected, making up the final sample. The municipalities analyzed had a total of 100,551 food establishments in 2022, of which 15,745 (15.7%) were located in the flooded area. Considering the extent and purpose of processing of the foods most frequently purchased in the establishments, 1,008 of those classified as in natura or minimally processed, 10,896 of ultra-processed, 3630 of mixed and 210 of super and hypermarkets were affected.

With regard to the proportion of each group of establishments in relation to the total number of establishments, the most affected group was that of establishments selling ultra-processed foods (69.2%) − the most frequent group in the state’s community food environment (p < 0.0001). The following stand out as the most affected categories of establishments: establishments that sell prepared foods for consumption (14.7%), in the group of ultra-processed foods; restaurants (14.6%), in the mixed group; fruit and vegetables (3.6%), in the group of in natura or minimally processed products; and supermarkets (1%) in the group of supermarkets and hypermarkets (p < 0.0001). A similar result was observed when analyzing the proportion of affected establishments in relation to the total number of affected establishments in the group according to the extent and purpose of processing, with supermarkets (73.3%), restaurants (63.1%), fruit and vegetables (55.7%), establishments that sell ready-to-eat foods (21.2%) standing out as the most affected categories within the groups of supermarkets and hypermarkets, mixed, in natura or minimally processed and ultra-processed. respectively (p < 0.0001). Finally, when observing the proportion of affected establishments in relation to the total number of establishments in each group of establishments, the group of establishments that sell in natura or minimally processed foods (17.1%) was the most affected among the other groups (p < 0.0001). In addition, statistically significant differences were also observed between the proportions of affected establishments in the categories of in natura or minimally processed and mixed groups, of which fishmongers (28.5%) and restaurants (17.6%) stand out as the most affected categories, respectively (p < 0.0001) (Table 1). 

**Table 1:** Characterization of food sales establishments affected by floods in municipalities of Rio Grande do Sul State, Brazil, 2024 (n = 92).

Type of establishment	Total (n)	Affected (n)	Affected in relation to the total (%)	Affected in relation to the Nova group * (%)	Affected in relation to category ** (%)
In natura or minimally processed	5,889	1,008	6.4	100.0	17.1 ^*** #^
Butcher shop	1,881	293	1.9	29.1	15.6
Produce	3,257	561	3.6 ^*** ##^	55.7 ^*** ##^	17.2
Dairy/Deli meats	456	70	0.4	6.9	15.4
Fishmonger	295	84	0.5	8.3	28.5 ^*** ##^
Ultra-processed foods	70,438	10,896	69.2 ^*** #^	100.0	15,5
Street food	7,887	1,281	8.1	11.8	16,2
Bars	2,074	330	2.1	3.0	15,9
Drinks	8,928	1,349	8.6	12.4	15,1
Canteen	331	56	0.4	0.5	16,9
Convenience	285	50	0.3	0.5	17,5
Sweets	791	123	0.8	1.1	15,5
Snack bar	14,289	2,281	14.5	20.9	16,0
Minimarket	14,624	2,174	13.8	20.0	14,9
Prepared for consumption	15,158	2,310	14.7 ^*** ##^	21.2 ^*** ##^	15,2
Retail/Unspecified	6,071	942	6.0	8.6	15,5
Mixed	22,581	3,630	23.1	100.0	16,1
Bakery production	6,538	895	5.7	24.7	13,7
Bakery resale	3,035	443	2.8	12.1	14,6
Restaurant	13,008	2,292	14.6 ^*** ##^	63.1 ^*** ##^	17.6 ^*** ##^
Supermarkets and hypermarkets	1,643	210	1.3	100.0	12,8
Supermarket	1,206	154	1.0 ^*** ##^	73.3 ^*** ##^	12,8
Hypermarket	437	57	0.4	27.1	13,0
Total	100,551	15,745	100.0	100.0	15,7

* Nova group includes “in natura or minimally processed”, “ultra-processed”, “mixed”, and also includes “super and hypermarkets”;

** Category refers to butchers, fruit and vegetable stores, dairy/cold cuts, fishmongers, street vendors, bars, canteens, convenience stores, sweets, snack bars, minimarkets, prepared for consumption, retail/unspecified, bakery production, bakery resale, restaurants, supermarkets, hypermarkets;

*** p < 0.001;

^#^ Comparison between the other Nova groups according to processing characteristics. The symbols are located in the groups and categories with the highest prevalence.

^##^ Comparison between the other categories of establishments in the same Nova group.

In addition, 31% of the census tracts of the affected cities were classified as food deserts. This proportion was higher in the flooded area (37.5%) when compared to the non-flooded area (28.5%) (p < 0.001) − data not shown in tables. 

Of the 92 municipalities analyzed, 27 stand out for having a proportion of affected food retail greater than 15.7%, which corresponds to the average percentage of food retail in the total sample. The total number of food retail in this subsample corresponds to 52% (n = 52,768) of the total sample. Among these municipalities, nine had more than 45% of the food retail affected: Santa Tereza (100%), Roca Sales (72%), Eldorado do Sul (68.8%), São Sebastião do Caí (62.1%), Picada Café (54.2%), Rolante (51.3%), Canoas (47.9%), Cruzeiro do Sul (47.4%), and São Leopoldo (47.3%) (Table 2).

**Table 2:** Characterization of food sales establishments affected by floods in the 27 most affected municipalities of Rio Grande do Sul State, Brazil, 2024.

Type of establishment	Total (n)	Affected (n)	Affected in relation to the total (%)	Affected in relation to the Nova group * (%)	Affected in relation to category ** (%)
In natura or minimally processed	2,949	864	6.3	100.0	29.3 ^*** #^
Butcher shop	941	249	1.8	28.8	26,5
Produce	1,626	483	3.5 ^*** ##^	55.9 ^*** ##^	29,7
Dairy/Deli meats	230	59	0.4	6.8	25,7
Fishmonger	152	73	0.5	8.4	48.0 ^*** ##^
Ultra-processed foods	35,821	9,437	68.6 ^*** #^	100.0	26.3
Street food	3,505	1,058	7.7	11.2	30.2 ^*** ##^
Bars	941	282	2.1	3.0	30.0
Drinks	4,337	1,169	8.5	12.4	27.0
Canteen	185	51	0.4	0.5	27.6
Convenience	178	45	0.3	0.5	25.3
Sweets	455	116	0.8	1.2	25.5
Snack bar	7,635	2,026	14.7	21.5 ^*** ##^	26.5
Minimarket	6,829	1,830	13.3	19.4	26.8
Prepared for consumption	8,615	2,032	14.8 ^*** ##^	21.5 ^*** ##^	23.6
Retail/Unspecified	3,141	828	6.0	8.8	26.4
Mixed	13,267	3,279	23.8	100.0	24.7
Bakery production	3,677	788	5.7	24.0	21.4
Bakery resale	1,634	391	2.8	11.9	23.9
Restaurant	7,956	2,100	15.3 ^*** ##^	64.0 *** ^##^	26.4 ^*** ##^
Supermarkets and hypermarkets	731	175	1.3	100.0	23.9
Supermarket	505	128	0.9 ^*** ##^	73.1 ^*** ##^	25.3
Hypermarket	226	47	0.3	26.9	20.8
Total	52,768	13,755	100.0	100.0	26.1

Note: among the 27 municipalities evaluated, with their percentage of affected commerce, are: Santa Tereza (100.0%), Roca Sales (72.0%), Eldorado do Sul (68.8%), São Sebastião do Caí (62.1%), Picada Café (54.2%), Rolante (51.3%), Canoas (47.9%), Cruzeiro do Sul (47.4%), São Leopoldo (47.3%), Bom Princípio (42.1%), Marques de Souza (41.7%), Muçum (36.7%), Estrela (34.7%), Igrejinha (31.8%), São Jerônimo (27.9%), Rio Grande (23.3%), Cerro Branco (22.2%), Lindolfo Collor (21.4%), Arroio do Meio (20.3%), Três Coroas (19.7%), Triunfo (18.8%), Guaíba (18.7%), Pelotas (17.5%), Porto Alegre (17.7%), and Encantado (17.4%). The symbols are located in the groups and categories with the highest prevalence.

* Nova group includes “in natura or minimally processed”, “ultra-processed”, “mixed”, and also includes “super and hypermarkets”;

** Category refers to butchers, fruit and vegetable stores, dairy/cold cuts, fishmongers, street vendors, bars, canteens, convenience stores, sweets, snack bars, minimarkets, prepared for consumption, retail/unspecified, bakery production, bakery resale, restaurants, supermarkets, hypermarkets.

*** p < 0.001;

# Comparison between the other Nova groups according to processing characteristics;

## Comparison between the other categories of establishments in the same Nova group.

**Figure 1:**
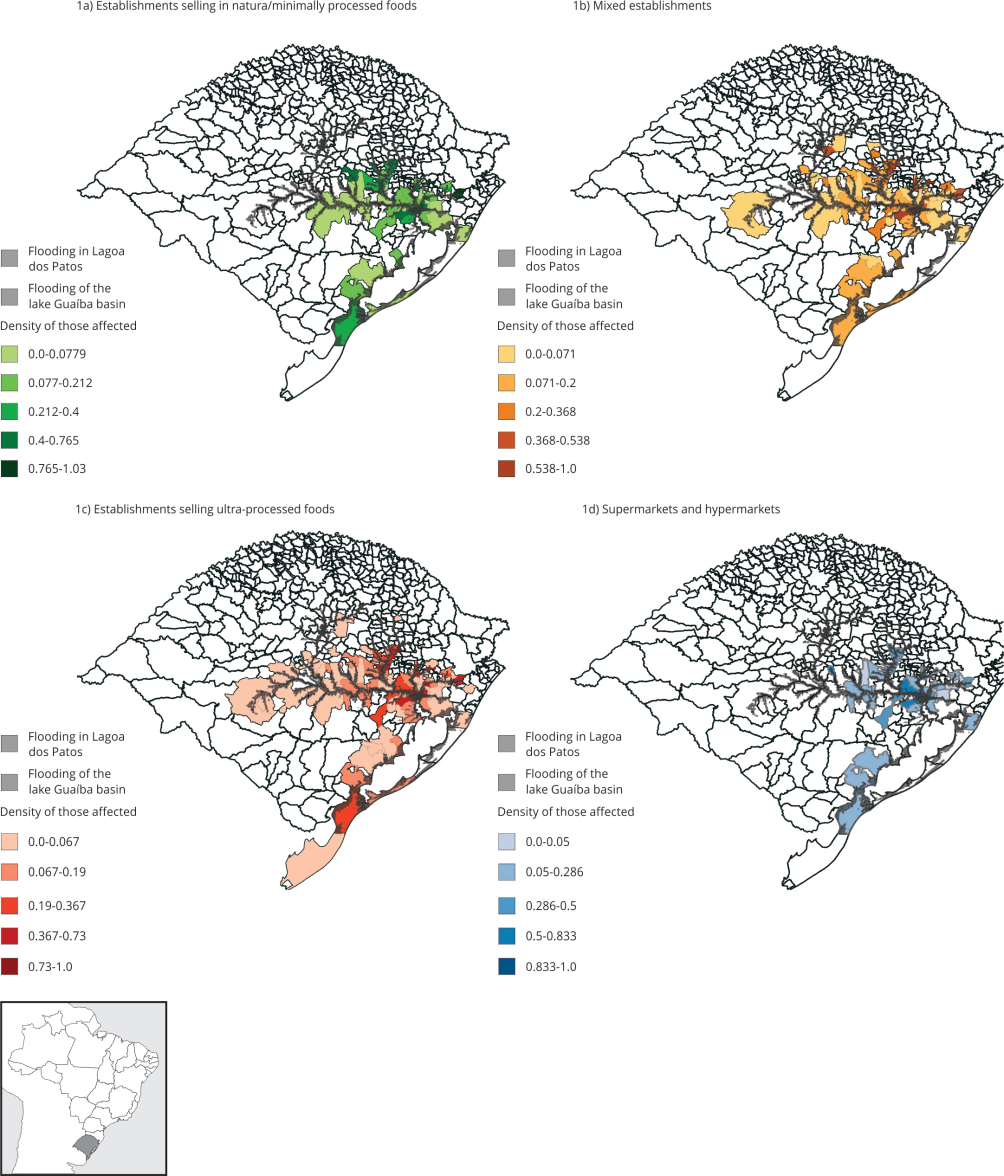
Impact of the flood on the food retail in municipalities in Rio Grande do Sul State, Brazil.

Figures 1a, 1b, 1c and 1d show the maps that describe the impact of the floods in the municipalities of Rio Grande do Sul whose food retail was affected by the floods, by group of establishments. The toned municipalities suffered losses, darker tones we denote a higher percentage impact in the groups of food establishments, highlighting that the most affected municipalities, in all groups, were north of lake Guaíba, as well as downstream of the Taquari river basin, to the north, south of Lagoa dos Patos (the largest in the state), and upstream of the Sinos river, to the east (highlighting the municipality of Rolante). The category of ultra-processed food establishments − the most prevalent in the state − has an impact distributed over more municipalities, expanding upstream of the Jacuí River and on the banks of the Vacacaí-mirim River, to the west, in addition to the regions mentioned above (Figure 1c). On the other hand, the losses to establishments in the group in natura or minimally processed and to super and hypermarkets are restricted to a smaller number of municipalities (Figures 1a and 1d). 

## Discussion

This study described the impact of the May 2024 floods on the food retail in the State of Rio Grande do Sul. Floods affected these establishments in a fifth of the state’s municipalities. In these municipalities, one sixth of the food retail was affected in some way, totaling more than 15 thousand establishments affected. In addition, some municipalities had half of their food retail affected. When analyzing the types and groups of establishments most affected, in natura food retail was proportionally the most affected among the four groups, with fruit and vegetable stores and fishmongers being the most affected. Businesses selling immediate food − restaurants, snack bars and stores that sell ready-to-eat food − were also greatly affected. Finally, food deserts were more frequent in the flooded census tracts. 

Floods have negative consequences on food systems that can lead to increased food insecurity through different pathways. Flooding can lead to losses or damage to crops and livestock, reducing the amount of food available, but it can also damage infrastructure such as roads, bridges, and storage facilities, preventing grown food from reaching food markets ^18^ ^,^ ^19^. In 2017, a riverine population in the Philippines, economically dependent on fishing, was affected by floods that aggravated the situation of food insecurity, with an increase in the prevalence of underweight during and after floods ^20^. Also in this sense, a study evaluated the effect of floods on food insecurity on the African continent between the years 2009 and 2020, demonstrating a 12% increase in food insecurity attributed to floods ^21^.

Similarly, floods in Rio Grande do Sul had a devastating impact on the agricultural rural households and can lead to local food shortages and possibly a price increase locally and in other regions of Brazil ^22^. In addition, the state’s infrastructure was greatly affected. Obstructions in roads and bridges have left areas isolated, making it difficult to transport food, even in areas not directly affected by the flooding. 

The community food environment is the link between food production and access to it by individuals, but studies evaluating the effect of climate disasters, such as floods, on the community food environment are still scarce. Rose et al. ^23^ evaluated the impacts of Hurricane Katrina, which devastated New Orleans, United States, on access to food retail. Access to supermarkets reduced by 42% compared to the pre-disaster scenario, and this reduction was greater in neighborhoods with a majority African-American population ^23^. In the United States, supermarkets are a marker of access to healthy food. In 2014, the city of Beni, in Bolivia, suffered from intense floods that destroyed both the food retail in the city, as well as the surrounding crops, a source of subsistence for a large part of the population; as a consequence, the scenario of food insecurity worsened in the region in the months following event ^24^. 

In like manner, our study showed a large impact of flooding on total establishments, with cities having half of all food retail affected, but also a greater impact on in natura or minimally processed food retail compared to the others. The decrease in food retail in general, and especially establishments that sell healthy food, can also increase the areas considered food deserts in the state. Food deserts are areas with no or low supply of healthy food. In this study, one-third of the flooded areas were already food deserts. An analysis carried out in 2020 in the city of Porto Alegre, one of the cities most affected by floods, showed that food deserts are also more frequent in areas of greater social vulnerability and greater presence of ethnic-racial minorities ^25^. Monitoring the community food environment, as well as the presence of food deserts, in this context is essential, since a community food environment with a low supply of food, especially healthy food, and with high food prices, contributes to the increase in food and nutrition insecurity, especially among populations that are already vulnerable ^26^ ^,^ ^27^. This is especially important in a scenario of increasing food and nutrition insecurity. In the state of Rio Grande do Sul, about 47% of the population was at some degree of food insecurity in 2022 ^28^. Added to this are the negative effects that this type of disaster has on the economy, with a possible increase in unemployment and impoverishment of the most vulnerable population. 

Knowing the types of establishments most affected is also essential to assess the impact of floods on the state’s food supply and plan focused public policies. The food retail for direct consumption, such as restaurants, snack bars, stores that sell ready-to-eat food were the most affected, this increased the difficulty in accessing meals during the most critical moments of the flood, when a large portion of the population was away from home; About 581 thousand people were left homeless at the height of crisis ^2^, or even, even at home, had access to water and electricity compromised. The fruit and vegetable trade was also greatly impacted. It should be noted that the largest fruit and vegetable supply center in the state, Rio Grande do Sul Supply Centers (CEASA, acronym in Portuguese), was also flooded, being out of operation for 40 days. This can make it even more difficult to access this food group, both in the short and long term. Finally, a fifth of the state's fishmongers were affected and, in the group of most affected municipalities, this figure rises to half. This makes it essential for the government to take a special look at the fishing population and also at the fish trade. In addition, these types of food retail are typically small enterprises that will have greater difficulty in reestablishing themselves than large supermarket chains. 

The restoration of the supply and community food environment after extreme weather events are imperative, in the prevention of food and nutrition insecurity, and can be achieved through actions and public policies aimed at ensuring access to healthy food, reducing socioeconomic vulnerability, promoting the resilience of communities, and strengthening local food systems. Among the actions and public policies proposed is the use of equipment that makes up the Brazilian National System of Food and Nutritional Security (SISAN, acronym in Portuguese), such as solidarity kitchens and popular restaurants as a means of facilitating access to quality meals, especially for the most vulnerable populations ^29^ ^,^ ^30^. In addition, the food supply can benefit from the strengthening and preparation of the Brazilian National Supply Company (CONAB, acronym in Portuguese) and Food Banks for this type of situation. In addition, the need to consolidate a national force for food and nutritional security is highlighted, to be activated in extreme weather events, in order to mobilize efforts to ensure healthy and quality food (e.g., creation of temporary kitchens to serve the population on an emergency basis); preparation of state and municipal food security plans that consider the climate emergency, strategically organizing the management of climate disaster scenarios, such as droughts and floods, with regard to the availability and access to food, in order to mitigate their consequences on food and nutritional security. 

Social and regional inequalities can also mark the resumption of food retail, given the heterogeneous distribution of the impact of floods in the different municipalities of the state, and this should be monitored, so as not to further aggravate the social inequalities in health already present in the state. In this sense, it is important to monitor the occurrence of food deserts in the long term. Future studies that assess the increase in food prices in the state, as well as the levels of food insecurity in the population are also essential to monitor the impacts of floods on the health and nutrition of the population. Granting tax incentives to small food traders, given that many have been impacted by this scenario, in order to enable the resumption of their commercial activities as soon as possible is a fundamental action.

### Limitations and strengths

The results of this study should be analyzed considering possible methodological limitations. The data used do not allow the evaluation of the extent of the damage caused by water in the establishment, they probably differ between each establishment. Damage caused by flooding or landslides in areas not adjacent to the rivers, even if resulting from floods, was also not accounted for. The data from the food retail used refer to the year 2022, as they are the most recent data available for analysis at the time of the emergency. Although changes in the community food environment between 2022 and 2024 cannot be ruled out, these are likely to be small and would not bias the results, since the changes tend not to show a systematic pattern, i.e., it is unlikely that any specific category or group of establishments or establishments in a single area will open or close. The database used does not include street markets and informal food trade. Outdoor markets are important points of acquisition of fruits and vegetables in Brazil. 

On the other hand, the data used are easy to obtain and analyze, making it possible to quickly describe the impact of floods on the food environment for all cities in the state, which is essential to support public policies that mitigate the effects of floods, in addition, it allows the monitoring of the restructuring of the food environment in the coming years. The subset of the most affected cities was also analyzed in order to assess whether the way in which the food retail was impacted was the same. To our knowledge, this is the work that investigates in depth the impact of an extreme weather event on the community food environment, analyzing the impact on all available food retail. 

## Conclusion

This study demonstrated the great impact of the floods of May 2024 on the community food environment of Rio Grande do Sul, especially in the group of food retail that sell in natura or minimally processed foods. In addition, the frequency of food deserts was higher in the affected areas. These results denote the importance of monitoring the reestablishment of the community food environment over time, considering possible differences in recovery capacity according to size, type, and group of establishments. Public food policies are fundamental in the restructuring of the state's food environment, these policies should focus on privileging the restructuring of businesses that are the basis for regular access to healthy food by the affected population.
