# Supplementary material for: Climate emergency and the food system: the impact of May floods on the community food environment of the Rio Grande do Sul State, Brazil
Source: Cad Saude Publica. 2025 May 26;41(4):e00130824. doi: 10.1590/0102-311XEN130824 (PMC12108468; doi:10.1590/0102-311XEN130824)
Supplement: Translation

# Translation: Emergência climática e o sistema alimentar: o impacto das enchentes de maio no ambiente alimentar comunitário do Rio Grande do Sul, Brasil

**DOI:** 10.1590/0102-311XPT130824

**Authors:** Lauren Yurgel da Silva, Elma Izze da Silva Magalhães, Júlio Celso Borello Vargas, Larissa Loures Mendes, Raquel Canuto

**Affiliations:** 1 Programa de Pós-graduação em Alimentação, Nutrição e Saúde, Universidade Federal do Rio Grande do Sul, Porto Alegre, Brasil.; 2 Programa de Pós-graduação em Planejamento Urbano e Regional, Universidade Federal do Rio Grande do Sul, Porto Alegre, Brasil.; 3 Departamento de Nutrição, Universidade Federal de Minas Gerais, Belo Horizonte, Brasil.

**Keywords:** Ambiente Alimentar, Mudança Climática, Enchentes

## Abstract

O objetivo deste estudo foi descrever o impacto das enchentes de maio de 2024 no ambiente alimentar comunitário do Rio Grande do Sul, Brasil. Trata-se de um estudo ecológico nos 92 municípios do Rio Grande do Sul que tiveram pelo menos um estabelecimento que comercializa alimentos atingido pelas enchentes. Os dados geográficos da inundação foram extraídos da base de dados do Instituto de Pesquisas Hidráulicas da Universidade Federal do Rio Grande do Sul e as informações dos estabelecimentos que comercializam alimentos obtidas da base de dados da Secretaria Especial da Receita Federal do Brasil (ano de 2022). Proporções de grupos/categorias de estabelecimentos afetados foram calculadas e comparadas por meio do teste qui-quadrado de Pearson. Do total de estabelecimentos dos municípios, 15,7% dos localizavam-se na área inundada. Além disso, 11 cidades tiveram mais de 40% do seu comércio inundado. Quanto à proporção de estabelecimentos afetados em relação ao total de cada grupo/categoria, o grupo de estabelecimentos que comercializam alimentos *in natura* ou minimamente processados (17,1%) foi o mais afetado (p < 0,0001); e as peixarias (28,5%) e os restaurantes (17,6%) foram as categorias mais afetadas dos grupos de *in natura* ou minimamente processados e mistos, respectivamente (p < 0,0001). Em conclusão, observou-se um grande impacto das enchentes no comércio de alimentos do Rio Grande do Sul, principalmente aqueles que trabalham com alimentos *in natura* ou minimamente processados, denotando a importância do monitoramento do restabelecimento do ambiente alimentar comunitário ao longo do tempo para garantia da segurança alimentar da população.

## Introdução

O Brasil passou por vários desastres ambientais na última década, incluindo os rompimentos das barragens de rejeitos de minério de Mariana (2015) e Brumadinho (2019), Minas Gerais, Brasil ^1^. Mas foi em maio de 2024 que o Rio Grande do Sul, estado mais meridional do Brasil com cerca de 11 milhões de habitantes, enfrentou um dos desastres climáticos mais significativos do país, tendo 95% de seus municípios afetados pelas maiores enchentes de sua história. Aproximadamente 2 milhões de pessoas foram afetadas, 581.638 foram deslocadas, 800 ficaram feridas e 178 mortes foram confirmadas ^2^.

As enchentes são consequência direta do superaquecimento dos oceanos, que alterou os padrões de chuvas no estado, aumentando a frequência das tempestades e o volume de precipitação ^3^. Esse tipo de evento climático extremo é cada vez mais frequente, e causa diversos prejuízos, incluindo a desestruturação dos sistemas alimentares ^4^ ^,^ ^5^ ^,^ ^6^ ^,^ ^7^. O sistema alimentar refere-se a um conjunto de elementos e atividades relacionadas à produção, processamento, distribuição, preparação e consumo de alimentos e os resultados dessas atividades ^8^. Ambiente alimentar pode ser definido como a interface do consumidor com o sistema alimentar, que abrange a disponibilidade, acessibilidade econômica, conveniência, promoção, qualidade e sustentabilidade de alimentos e bebidas em espaços selvagens, cultivados e construídos, influenciados pelo ambiente sociocultural, político e pelos ecossistemas nos quais estão inseridos ^9^. O ambiente alimentar comunitário é uma das dimensões do sistema alimentar e diz respeito à disponibilidade (distribuição, número e tipo) e acessibilidade dos estabelecimentos comerciais de alimentos ^10^.

O ambiente alimentar comunitário pode ser particularmente afetado por enchentes devido aos danos causados às infraestruturas e espaços físicos de estabelecimentos nos quais os alimentos são produzidos, estocados e comercializados ^4^, reduzindo assim a disponibilidade de produtos alimentícios e dificultando o acesso físico e econômico da população aos diversos tipos de alimentos ^6^ ^,^ ^7^. Este cenário contribui para o aumento da insegurança alimentar e nutricional, reduzindo a oferta de alimentos *in natura* ou minimamente processados e favorecendo o consumo de ultraprocessados, especialmente entre populações vulneráveis ^11^ ^,^ ^12^. Tais fatores estão no cerne da sindemia global, constituída pelas pandemias da desnutrição, obesidade e mudanças climáticas, que interagem sinergicamente e compartilham determinantes sociais comuns, caracterizando um panorama desafiador em escala global ^12^.

Dado que os eventos climáticos extremos podem impactar a garantia da segurança alimentar e nutricional das populações afetadas, torna-se imperativo avaliar o impacto das enchentes no ambiente alimentar comunitário. Assim, o objetivo deste trabalho foi descrever o impacto das enchentes que ocorreram em maio de 2024 no ambiente alimentar comunitário do Rio Grande do Sul.

## Métodos

Trata-se de um estudo ecológico que teve como unidade de análise municípios do Rio Grande do Sul que tiveram os comércios de alimentos atingidos pelas enchentes de maio de 2024. O Estado do Rio Grande do Sul, localizado na Região Sul do país, possui uma área geográfica de 281.707,15km^2^, com 497 municípios e uma população de 10.882.965 habitantes ^13^. A amostra analisada no presente estudo foi composta pelos municípios que tiveram pelo menos um estabelecimento que comercializa alimentos atingido pelas enchentes, ou seja, que estava localizado na faixa de inundação observada por análise geoespacial. Desta amostra, foi analisada uma subamostra dos municípios que tiveram uma proporção de estabelecimentos que comercializam alimentos atingidos maior que o percentual médio observado na amostra total, para melhor compreender o impacto da enchente nas áreas com uma maior proporção de estabelecimentos que comercializam alimentos afetados. Essa subamostra foi selecionada calculando-se o percentual médio de estabelecimentos que comercializam alimentos atingidos da amostra total e, em seguida, identificando-se aqueles municípios cujo percentual era superior a esse valor.

Os dados geográficos da inundação foram extraídos da base de dados do Instituto de Pesquisas Hidráulicas da Universidade Federal do Rio Grande do Sul (UFRGS), de acesso disponível ao público na plataforma *Cheias no Rio Grande do Sul - Base de Dados e informações geográficas na Região Hidrográfica do Lago Guaíba e na Lagoa dos Patos em 2024*, pelo ArcGIS StoryMaps ^14^. Os mapas disponibilizados mostram as manchas da área de inundação na região hidrográfica do lago Guaíba e da lagoa dos Patos, captadas por satélite do sensor Sentinel-2 nos dias 6 a 8 de maio de 2024.

As informações dos estabelecimentos que comercializam alimentos foram obtidas da base de dados da Secretaria Especial da Receita Federal do Brasil (RFB), que condensa os dados dos Cadastro Nacional da Pessoa Jurídica (CNPJ) ativos, referentes ao ano de 2022. A base de dados continha informações a respeito do endereço, nome, porte da empresa, tipo principal de atividade exercida e classificação conforme a Classificação Nacional das Atividades Econômicas (CNAE). Essa classificação deriva de uma padronização das atividades comerciais exercidas por meio de códigos, que totalizaram 19 tipos diferentes de estabelecimentos. Com os endereços, foram extraídas as coordenadas geográficas por meio do API de georreferenciamento do Google Maps (https://www.google.com/maps/), via extensão do Awesome Table - Talarian (https://awesome-table.com/), uma extensão gratuita que viabiliza a comunicação entre o API de georreferenciamento e uma planilha do Google Sheets (https://workspace.google.com/products/sheets/). A correção de erros de georreferenciamento foi realizada utilizando-se o software para sistemas de informação geográfica QGIS 3.34.7 (https://qgis.org/en/site/), no qual os pontos com erros foram visualizados e corrigidos por meio de novo georreferenciamento.

Após, os estabelecimentos foram agrupados conforme a metodologia proposta pela Câmara Interministerial de Segurança Alimentar e Nutricional ^15^, que classifica os estabelecimentos de acordo com a extensão e propósito de processamento dos alimentos mais frequentemente adquiridos pela população brasileira em cada categoria de estabelecimento. Essa metodologia deriva da classificação Nova ^16^. Os estabelecimentos foram então classificados nos seguintes grupos: (1) estabelecimentos que comercializam principalmente alimentos *in natura* ou minimamente processados, como hortifrutigranjeiros, açougues e peixarias; (2) estabelecimentos que vendem predominantemente alimentos ultraprocessados, como bares, lanchonetes, lojas de conveniência e de doces; e (3) estabelecimentos mistos, que vendem uma mescla das demais categorias de alimentos, como padarias e restaurantes. Supermercados e hipermercados foram analisados separadamente devido à grande variedade de alimentos comercializados e pela falta de consenso na literatura sobre sua real influência no perfil de compra da população ^17^.

Por meio de análises geoespaciais no QGIS 3.34.7, realizou-se a interpolação das áreas de inundação e dos estabelecimentos que comercializam alimentos georreferenciados, sendo considerados como afetados os estabelecimentos que comercializavam alimentos que se encontravam dentro da área de inundação.

As análises estatísticas foram conduzidas no software Stata versão 14.0 (https://www.stata.com). Os dados analisados foram descritos por meio de frequências absolutas e relativas, considerando os conceitos de categoria de estabelecimentos (tipos de estabelecimento, como açougues, lanchonetes, restaurantes, supermercados etc.) e grupos de estabelecimentos de acordo com a classificação Nova (*in natura* ou minimamente processados, mistos e ultraprocessados). Foram calculadas três diferentes medidas relativas à proporção de categorias de estabelecimentos afetados: em relação ao total de estabelecimentos; em relação ao total de estabelecimentos afetados do grupo; e em relação ao total de estabelecimentos da própria categoria.

Além disso, foi identificado o percentual de setores censitários (unidade de análise) classificados como desertos alimentares nas cidades que tiveram os estabelecimentos que comercializam alimentos afetados, da seguinte forma: foi calculada a densidade de estabelecimentos saudáveis (*in natura* ou minimamente processados, mistos e supermercados e hipermercados) a cada 10 mil habitantes. Aqueles setores com densidade menor ou igual ao percentil 25 para foram classificados como desertos alimentares ^15^. O teste qui-quadrado de Pearson foi utilizado para comparar as proporções analisadas, considerando-se um nível de 5% de significância.

Para a representação visual do impacto da enchente no comércio de alimentos, os percentuais de estabelecimentos afetados por categoria em cada município atingido foram representados em mapas com graduação por cores, usando *Quebras de Jenks* em cinco tons, em que tons mais claros indicam um menor impacto da enchente, e tons mais escuros representam um maior impacto.

## Resultados

Dos 497 municípios do estado, 191 foram afetados pela inundação e 92 tiveram seus estabelecimentos que comercializam alimentos afetados, compondo a amostra final. Os municípios analisados possuíam ao todo 100.551 estabelecimentos que comercializam alimentos em 2022, dos quais 15.745 (15,7%) localizavam-se na área inundada. Considerando a extensão e propósito de processamento dos alimentos mais frequentemente adquiridos nos estabelecimentos, foram atingidos 1.008 daqueles classificados como comércio de *in natura* ou minimamente processados, 10.896 dos comércios de ultraprocessados, 3.630 dos de mistos e 210 dos super e hipermercados.

No que diz respeito à proporção de cada grupo de estabelecimentos em relação ao total, o grupo mais afetado foi o dos estabelecimentos de venda de alimentos dos ultraprocessados (69,2%) - o mais frequente no ambiente alimentar comunitário do estado (p < 0,0001). Destacam-se como as categorias de estabelecimentos mais afetadas: estabelecimentos que vendem alimentos preparados para o consumo (14,7%), no grupo dos ultraprocessados; restaurantes (14,6%), no grupo dos mistos; os hortifrútis (3,6%), no grupo dos *in natura* ou minimamente processados; e supermercados (1%), no grupo de supermercados e hipermercados (p < 0,0001). Resultado similar foi observado quando analisada a proporção de estabelecimentos afetados em relação ao total de estabelecimentos afetados do grupo de acordo com a extensão e propósito de processamento, destacando-se os supermercados (73,3%), restaurantes (63,1%), hortifrútis (55,7%), estabelecimentos que comercializam alimentos prontos para o consumo (21,2%) como as categorias mais afetadas dentro dos grupos de supermercados e hipermercados, mistos, *in natura* ou minimamente processados e ultraprocessados, respectivamente (p < 0,0001). Por fim, ao observarmos a proporção de estabelecimentos afetados em relação ao total de cada grupo de estabelecimentos, o grupo dos comércios de alimentos *in natura* ou minimamente processados (17,1%) foi o mais afetado dentre os demais grupos (p < 0,0001). Além disso, também foram observadas diferenças estatisticamente significativas entre as proporções de estabelecimentos afetados nas categorias dos grupos de *in natura* ou minimamente processados e mistos, dos quais destacam-se as peixarias (28,5%) e os restaurantes (17,6%) como as categorias mais afetadas, respectivamente (p < 0,0001) (Tabela 1).

**Tabela 1:** Caracterização dos estabelecimentos de vendas de alimentos afetados pelas enchentes em municípios do Rio Grande do Sul, Brasil, 2024 (n = 92).

Tipo de estabelecimento	Total (n)	Afetados (n)	Afetados em relação ao total (%)	Afetados em relação ao grupo da Nova *(%)	Afetados em relação à categoria ** (%)
*In natura* ou minimamente processados	5.889	1.008	6,4	100,0	17,1 * ^#^
Açougue	1.881	293	1,9	29,1	15,6
Hortifrúti	3.257	561	3,6 *** ^##^	55,7 *** ^##^	17,2
Laticínios/Frios	456	70	0,4	6,9	15,4
Peixaria	295	84	0,5	8,3	28,5 *** ^##^
Ultraprocessados	70.438	10.896	69,2 *** ^#^	100,0	15,5
Ambulante	7.887	1.281	8,1	11,8	16,2
Bares	2.074	330	2,1	3,0	15,9
Bebidas	8.928	1.349	8,6	12,4	15,1
Cantina	331	56	0,4	0,5	16,9
Conveniência	285	50	0,3	0,5	17,5
Doces	791	123	0,8	1,1	15,5
Lanchonete	14.289	2.281	14,5	20,9	16,0
Minimercado	14.624	2.174	13,8	20,0	14,9
Preparados para consumo	15.158	2.310	14,7 *** ^##^	21,2 *** ^##^	15,2
Varejo/Não especificado	6.071	942	6,0	8,6	15,5
Mistos	22.581	3.630	23,1	100,0	16,1
Padaria produção	6.538	895	5,7	24,7	13,7
Padaria revenda	3.035	443	2,8	12,1	14,6
Restaurante	13.008	2.292	14,6 *** ^##^	63,1 *** ^##^	17,6 *** ^##^
Supermercados e hipermercados	1.643	210	1,3	100,0	12,8
Supermercado	1.206	154	1 *** ^##^	73,3 *** ^##^	12,8
Hipermercado	437	57	0,4	27,1	13,0
Total	100.551	15.745	100,0	100,0	15,7

* Grupos classificados de acordo com a Nova compreendem “*in natura* ou minimamente processados”, “ultraprocessados”, “mistos” e “super e hipermercados”;

** Categoria refere-se a açougue, hortifrúti, laticínios/frios, peixaria, ambulante, bares, cantina, conveniência, doces, lanchonete, minimercado, preparados para consumo, varejo/não especificado, padaria produção, padaria revenda, restaurante, supermercado, hipermercado;

*** p < 0,001;

^#^ Comparação entre os demais grupos da Nova de acordo com as características de processamento. Os símbolos estão localizados nos grupos e nas categorias com maior prevalência;

^##^ Comparação entre as demais categorias de estabelecimento no mesmo grupo da Nova.

Além disso, 31% dos setores censitários das cidades afetadas foram classificados como desertos alimentares. Essa proporção foi maior na área inundada (37,5%), quando comparada com a área não inundada (28,5%) (p < 0,001) - dados não apresentados em tabelas.

Dos 92 municípios analisados, 27 se destacam por terem uma proporção de estabelecimentos que comercializam alimentos afetados maior que 15,7%, que corresponde ao percentual médio de estabelecimentos que comercializam alimentos na amostra total. O total de estabelecimentos que comercializam alimentos dessa subamostra corresponde a 52% (n = 52.768) da amostra total. Dentre esses municípios, nove tiveram mais de 45% dos estabelecimentos que comercializam alimentos afetados: Santa Tereza (100%), Roca Sales (72%), Eldorado do Sul (68,8%), São Sebastião do Caí (62,1%), Picada Café (54,2%), Rolante (51,3%), Canoas (47,9%), Cruzeiro do Sul (47,4%) e São Leopoldo (47,3%) (Tabela 2).

**Tabela 2:** Caracterização dos estabelecimentos de vendas de alimentos afetados pelas enchentes nos 27 municípios mais atingidos do Rio Grande do Sul, Brasil, 2024.

Tipo de estabelecimento	Total (n)	Afetados (n)	Afetados em relação ao total (%)	Afetados em relação ao grupo da NOVA * (%)	Afetados em relação à categoria ** (%)
*In natura* ou minimamente processados	2.949	864	6,3	100,0	29,3 *** ^#^
Açougue	941	249	1,8	28,8	26,5
Hortifrúti	1.626	483	3,5 *** ^##^	55,9 *** ^##^	29,7
Laticínios/Frios	230	59	0,4	6,8	25,7
Peixaria	152	73	0,5	8,4	48 *** ^##^
Ultraprocessados	35.821	9.437	68,6 *** ^#^	100,0	26,3
Ambulante	3.505	1.058	7,7	11,2	30,2 *** ^##^
Bares	941	282	2,1	3,0	30,0
Bebidas	4.337	1.169	8,5	12,4	27,0
Cantina	185	51	0,4	0,5	27,6
Conveniência	178	45	0,3	0,5	25,3
Doces	455	116	0,8	1,2	25,5
Lanchonete	7.635	2.026	14,7	21,5 *** ^##^	26,5
Minimercado	6.829	1.830	13,3	19,4	26,8
Preparados para consumo	8.615	2.032	14,8 *** ^##^	21,5 *** ^##^	23,6
Varejo/Não especificado	3.141	828	6,0	8,8	26,4
Mistos	13.267	3.279	23,8	100,0	24,7
Padaria produção	3.677	788	5,7	24,0	21,4
Padaria revenda	1.634	391	2,8	11,9	23,9
Restaurante	7.956	2.100	15,3 *** ^##^	64 *** ^##^	26,4 *** ^##^
Supermercados e hipermercados	731	175	1,3	100,0	23,9
Supermercado	505	128	0,9 *** ^##^	73,1 *** ^##^	25,3
Hipermercado	226	47	0,3	26,9	20,8
Total	52.768	13.755	100,0	100,0	26,1

Nota: dentre os 27 municípios avaliados, com o seu percentual de comércio afetado, estão: Santa Tereza (100%), Roca Sales (72%), Eldorado do Sul (68,8%), São Sebastião do Caí (62,1%), Picada Café (54,2%), Rolante (51,3%), Canoas (47,9%), Cruzeiro do Sul (47,4%), São Leopoldo (47,3%), Bom Princípio (42,1%), Marques de Souza (41,7%), Muçum (36,7%), Estrela (34,7%), Igrejinha (31,8%), São Jerônimo (27,9%), Rio Grande (23,3%), Cerro Branco (22,2%), Lindolfo Collor (21,4%), Arroio do Meio (20,3%), Três Coroas (19,7%), Triunfo (18,8%), Guaíba (18,7%), Pelotas (17,5%), Porto Alegre (17,7%), e Encantado (17,4%). Os símbolos estão localizados nos grupos categorias com maior prevalência.

* Grupo classificados de acordo com a Nova compreendem “in natura ou minimamente processados”, “ultraprocessados”, “mistos” e “super e hipermercados”;

** Categoria refere-se a açougue, hortifrúti, laticínios/frios, peixaria, ambulante, bares, cantina, conveniência, doces, lanchonete, minimercado, preparados para consumo, varejo/não especificado, padaria produção, padaria revenda, restaurante, supermercado, hipermercado;

*** p < 0,001;

# Comparação entre os demais grupos de estabelecimentos de acordo com as características de processamento.

## Comparação entre as demais categorias de estabeleciment.

Nas Figuras 1a, 1b, 1c e 1d, estão apresentados os mapas que descrevem o impacto das enchentes nos municípios do Rio Grande do Sul cujos estabelecimentos que comercializam alimentos foram atingidos pelas enchentes, por grupo de estabelecimentos. Os municípios tonalizados sofreram perdas. Tons mais escuros denotam maior impacto percentual nos grupos de estabelecimentos de venda de alimentos, destacando que os municípios mais afetados, em todos os grupos, estavam ao norte do lago Guaíba, bem como a jusante da bacia do rio Taquari, ao norte, ao sul da lagoa dos Patos (a maior do estado), e a montante do rio dos Sinos, a leste (a destacar o Município de Rolante). O grupo de estabelecimentos de venda de ultraprocessados - a mais prevalente no estado - apresenta um impacto distribuído por mais municípios, se expandindo a montante do rio Jacuí e às margens do rio Vacacaí-Mirim, a oeste, além das regiões anteriormente citadas (Figura 1c). Por outro lado, as perdas de estabelecimentos do grupo dos *in natura* ou minimamente processados e de super e hipermercados fica restrita a um menor número de municípios (Figura 1a e 1d).

**Figura 1:** Impacto da enchente nos estabelecimentos que comercializam alimentos nos municípios do Rio Grande do Sul, Brasil.

## Discussão

Este estudo descreveu o impacto das enchentes de maio de 2024 nos estabelecimentos que comercializam alimentos do Estado do Rio Grande do Sul. As inundações afetaram estes estabelecimentos em um quinto dos municípios do estado. Nesses municípios, um sexto dos estabelecimentos que comercializam alimentos foi afetado de alguma forma, totalizando mais de 15 mil. Além disso, alguns municípios tiveram metade dos comércios de alimentos afetados. Quando analisadas as categorias e grupos de estabelecimentos mais afetados, aqueles que comercializam alimentos *in natura* ou minimamente processados foram proporcionalmente os mais afetados entre os quatro grupos, sendo os estabelecimentos que comercializam frutas e verduras e as peixarias os mais afetados. Os que comercializam alimentos de consumo imediato - restaurantes, lanchonetes e lojas que vendem alimentos prontos para o consumo - foram também bastante afetados. Por fim, desertos alimentares foram mais frequentes nos setores censitários inundados.

Enchentes têm consequências negativas nos sistemas alimentares que podem levar ao aumento da insegurança alimentar por diferentes caminhos. As inundações podem levar a perdas ou danos às culturas e ao gado, reduzindo a quantidade de alimentos disponíveis, mas também podem danificar infraestruturas, como estradas, pontes e instalações de armazenamento, impedindo que o alimento cultivado chegue aos mercados de alimentos ^18^ ^,^ ^19^. Em 2017 uma população ribeirinha das Filipinas, economicamente dependente da pesca, foi afetada por enchentes que agravaram a situação de insegurança alimentar, com aumento da prevalência de baixo peso durante e após as enchentes ^20^. Ainda nesse sentido, um estudo avaliou o efeito das enchentes na insegurança alimentar no continente africano entre os anos de 2009 e 2020, demonstrando um aumento de 12% na insegurança alimentar atribuída às inundações ^21^.

De forma semelhante, as inundações no Rio Grande do Sul tiveram um impacto devastador no setor agropecuário, com 4.509 instalações agropecuárias afetadas. Os danos a essas instalações não apenas interrompem a produção agrícola e pecuária, mas também ameaçam a sustentabilidade econômica de muitas famílias rurais e podem levar a uma escassez local de produtos alimentares e possivelmente um aumento de preço local e em outras regiões do Brasil ^22^. Além disso, a infraestrutura do estado foi bastante afetada. As obstruções em estradas e pontes deixaram áreas isoladas, dificultando o transporte de alimentos, mesmo nas áreas não afetadas diretamente pela inundação.

O ambiente alimentar comunitário é o elo entre a produção dos alimentos e o acesso pelos indivíduos, porém estudos que avaliem o efeito de desastres climáticos, como enchentes, no ambiente alimentar comunitário ainda são escassos. Rose et al. ^23^ avaliaram os impactos do furacão Katrina, que assolou Nova Orleans (Estado Unidos) em 2005, no acesso a estabelecimentos que comercializam alimentos. O acesso a supermercados reduziu em 42% comparado ao cenário anterior ao desastre, e essa redução foi maior nos bairros de população majoritariamente afro-americana ^23^. Nos Estados Unidos, os supermercados são um marcador de acesso a alimentos saudáveis. Já em 2014, a cidade de Beni, na Bolívia, sofreu com enchentes intensas que destruíram tanto os estabelecimentos que comercializavam alimentos na cidade, quanto as lavouras no entorno, fonte de subsistência de boa parte da população; como consequência, o cenário de insegurança alimentar se agravou na região nos meses subsequentes ao evento ^24^.

Da mesma forma, o nosso estudo demonstrou um grande impacto das enchentes no total de estabelecimentos, com cidades tendo metade dos estabelecimentos que comercializam alimentos afetada, mas também um maior impacto nos estabelecimentos que comercializam alimentos *in natura* ou minimamente processados em relação aos demais. A diminuição dos estabelecimentos que comercializam alimentos no geral, e principalmente daqueles que comercializam alimentos saudáveis, também pode aumentar as áreas consideradas desertos alimentares no estado. Desertos alimentares são áreas com nenhuma ou baixa oferta de alimentos saudáveis. Neste estudo, um terço das áreas inundadas já eram desertos alimentares. Análise feita em 2020, na cidade de Porto Alegre (Rio Grande do Sul), uma das cidades mais afetadas pelas enchentes, demonstrou que os desertos alimentares também são mais frequentes em áreas de maior vulnerabilidade social e maior presença de minorias étnico-raciais ^25^. Monitorar o ambiente alimentar comunitário, bem como a presença de desertos alimentares, nesse contexto, é fundamental, uma vez que ambiente alimentar comunitário com baixa oferta de alimentos, principalmente os saudáveis, e com elevado preço de alimentos, contribui para o aumento da insegurança alimentar e nutricional, principalmente entre as populações já vulnerabilizadas ^26^ ^,^ ^27^. Isso é especialmente importante num cenário de aumento da insegurança alimentar e nutricional. No Estado do Rio Grande do Sul, cerca de 47% da população encontrava-se em algum grau de insegurança alimentar em 2022 ^28^. Somam-se a isso os efeitos negativos que esse tipo de desastre tem sobre a economia, com possível aumento do desemprego e empobrecimento da população mais vulnerável.

Conhecer as categorias de estabelecimentos mais afetados também é fundamental para avaliar o impacto das enchentes no abastecimento de alimentos do estado e planejar políticas públicas focalizadas. Os estabelecimentos que comercializam alimentos para consumo direto, como restaurantes, lanchonetes, lojas que vendem alimentos prontos para o consumo foram os mais afetados. Isso aumentou a dificuldade em acessar refeições durante os momentos mais críticos do alagamento, quando uma grande parcela da população estava fora de casa; cerca de 581 mil pessoas ficaram desabrigadas no ápice da crise ^2^, ou ainda, mesmo em casa, estava com acesso a água e luz comprometidos. O comércio de frutas e verduras também foi bastante impactado. Destaca-se que a maior central de abastecimento de hortifrúti do estado (CEASA, Centrais de Abastecimento do Rio Grande do Sul), também foi alagada, ficando sem funcionamento por 40 dias. Isso pode tornar mais difícil ainda o acesso a esse grupo de alimentos, no curto e no longo prazo. Por fim, um quinto das peixarias do estado foram afetadas e, no grupo de municípios mais afetados, esse dado sobe para a metade. Isso torna fundamental um olhar especial do governo para a população de pescadores e também para o comércio de pescado. Além disso, esses estabelecimentos que comercializam alimentos são tipicamente de pequenos empreendimentos que terão maior dificuldade em se restabelecer do que grandes redes de supermercados.

Nesse sentido, é imperativo restabelecer o abastecimento e o ambiente alimentar comunitário após eventos climáticos extremos para prevenir a insegurança alimentar e nutricional. Isso pode ser alcançado por meio de ações e políticas públicas direcionadas para a garantia do acesso a alimentos saudáveis, redução da vulnerabilidade socioeconômica, promoção da resiliência das comunidades e fortalecimento de sistemas alimentares locais. Dentre as ações e políticas públicas, propõe-se a utilização de equipamentos que compõem o Sistema Nacional de Segurança Alimentar e Nutricional (SISAN), como as cozinhas solidárias e os restaurantes populares como meios de facilitar o acesso a refeições de qualidade, especialmente às populações mais vulneráveis ^29^ ^,^ ^30^. Além disso, o abastecimento de alimentos pode ser beneficiado pelo fortalecimento e preparação da Companhia Nacional de Abastecimento (CONAB) e Bancos de Alimentos para esse tipo de situação. Além disso, destaca-se a necessidade de consolidação de uma força nacional de segurança alimentar e nutricional, a ser acionada em eventos climáticos extremos, visando mobilizar esforços para garantir uma alimentação saudável e de qualidade (ex.: criação de cozinhas temporárias para atender à população em caráter emergencial) e elaboração de planos estadual e municipal de segurança alimentar que consideram a emergência climática, organizando de maneira estratégica a gestão de cenários de desastres climáticos, como secas e enchentes, no que tange a disponibilidade e acesso aos alimentos, a fim de mitigar suas consequências na segurança alimentar nutricional.

Por fim, desigualdades sociais e regionais também podem marcar a retomada dos estabelecimentos, dada a distribuição heterogênea do impacto das enchentes nos diferentes municípios do estado, e isso deve ser monitorado, para não agravar ainda mais as desigualdades sociais em saúde já presentes no estado. Nesse sentido, é importante que se monitore a ocorrência de desertos alimentares a longo prazo. Estudos futuros que avaliem o aumento dos preços nos alimentos no estado, bem como os níveis de insegurança alimentar na população, também são fundamentais para monitorar os impactos das enchentes na saúde e nutrição da população. Concessão de incentivos fiscais para pequenos comerciantes de alimentos, dado que muitos foram impactados com esse cenário, a fim de viabilizar a retomada de suas atividades comerciais o mais rapidamente possível, é uma ação fundamental.

### Limitações e pontos fortes

Os resultados desse trabalho devem ser analisados considerando possíveis limitações metodológicas. Os dados utilizados não permitem avaliar a extensão do dano causado pela água no estabelecimento; provavelmente, eles diferem entre cada estabelecimento. Também não foram contabilizados os danos causados por alagamentos ou deslizamentos em áreas não adjacentes aos rios, ainda que consequentes às enchentes. Os dados dos estabelecimentos que comercializam alimentos utilizados são referentes ao ano de 2022, por serem os mais recentes disponíveis para análise no momento da emergência. Embora mudanças no ambiente alimentar comunitário entre os anos de 2022 e 2024 não possam ser descartadas, essas provavelmente são pequenas e não enviesariam os resultados, uma vez que as mudanças tendem a não apresentar um padrão sistemático, ou seja, é pouco provável a abertura ou fechamento de alguma categoria ou grupo específico de estabelecimento ou de estabelecimentos em uma única área. A base de dados utilizada não inclui feiras livres e comércio informal de alimentos. As feiras livres são importantes pontos de aquisição de frutas e verduras no Brasil.

Por outro lado, os dados utilizados são de fácil obtenção e análise, possibilitando a descrição do impacto das enchentes no ambiente alimentar de forma rápida para todas as cidades do estado, o que é fundamental para embasar políticas públicas que mitiguem os efeitos das enchentes, além disso, permitem o monitoramento da reestruturação do ambiente alimentar nos próximos. Também foi analisado o subconjunto das cidades mais afetadas a fim de avaliar se a forma como os estabelecimentos que comercializam alimentos foram impactados era a mesma. Que seja do nosso conhecimento, esse é o trabalho que investiga de forma mais profunda o impacto de um evento climático extremo no ambiente alimentar comunitário, analisando o impacto em todos os estabelecimentos que comercializam alimentos disponíveis.

## Conclusão

Este estudo demonstrou o grande impacto das enchentes de maio de 2024 no ambiente alimentar comunitário do Rio Grande do Sul, principalmente no grupo de estabelecimentos que comercializam alimentos *in natura* ou minimamente processados. Além disso, a frequência de desertos alimentares foi maior nas áreas afetadas. Esses resultados denotam a importância do monitoramento do restabelecimento do ambiente alimentar comunitário ao longo do tempo, considerando possíveis diferenças na capacidade de recuperação de acordo com o tamanho, categoria e grupo de estabelecimento. Políticas públicas de alimentação são fundamentais na reestruturação do ambiente alimentar do estado. Essas políticas devem ter foco em privilegiar a reestruturação dos estabelecimentos que são base para o acesso regular aos alimentos saudáveis pela população atingida.
